# Variable Classification of Drug-Intoxication Suicides across US States: A Partial Artifact of Forensics?

**DOI:** 10.1371/journal.pone.0135296

**Published:** 2015-08-21

**Authors:** Ian R. H. Rockett, Gerald R. Hobbs, Dan Wu, Haomiao Jia, Kurt B. Nolte, Gordon S. Smith, Sandra L. Putnam, Eric D. Caine

**Affiliations:** 1 Injury Control Research Center and Department of Epidemiology, West Virginia University School of Public Health, Morgantown, West Virginia, United States of America; 2 Department of Statistics, West Virginia University, Morgantown, West Virginia, United States of America; 3 Department of Psychology, Guangdong Medical College, Dongguan, Guandong, China and Department of Social Medicine, Zhejiang University School of Medicine, Hangzhou, China; 4 Department of Biostatistics, Mailman School of Public Health, and School of Nursing, Columbia University, New York, New York, United States of America; 5 Office of the Medical Investigator – Department of Pathology, University of New Mexico School of Medicine, Albuquerque, New Mexico, United States of America; 6 Department of Epidemiology and Public Health, University of Maryland, Baltimore, Maryland, United States of America; 7 Social Solutions International Inc., Silver Spring, Maryland, United States of America; 8 Injury Control Research Center for Suicide Prevention and Department of Psychiatry, University of Rochester Medical Center, Rochester, New York, United States of America; Temple University School of Medicine, UNITED STATES

## Abstract

**Background:**

The 21^st^-century epidemic of pharmaceutical and other drug-intoxication deaths in the United States (US) has likely precipitated an increase in misclassified, undercounted suicides. Drug-intoxication suicides are highly prone to be misclassified as accident or undetermined. Misclassification adversely impacts suicide and other injury mortality surveillance, etiologic understanding, prevention, and hence clinical and public health policy formation and practice.

**Objective:**

To evaluate whether observed variation in the relative magnitude of drug-intoxication suicides across US states is a partial artifact of the scope and quality of toxicological testing and type of medicolegal death investigation system.

**Methods:**

This was a national, state-based, ecological study of 111,583 drug-intoxication fatalities, whose manner of death was suicide, accident, or undetermined. The proportion of (nonhomicide) drug-intoxication deaths classified by medical examiners and coroners as suicide was analyzed relative to the proportion of death certificates citing one or more specific drugs and two types of state death investigation systems. Our model incorporated five sociodemographic covariates. Data covered the period 2008–2010, and derived from NCHS’s Multiple Cause-of-Death public use files.

**Results:**

Across states, the proportion of drug-intoxication suicides ranged from 0.058 in Louisiana to 0.286 in South Dakota and the rate from 1 per 100,000 population in North Dakota to 4 in New Mexico. There was a low correlation between combined accident and undetermined drug-intoxication death rates and corresponding suicide rates (Spearman’s rho = 0.38; p<0.01). Citation of 1 or more specific drugs on the death certificate was positively associated with the relative odds of a state classifying a nonhomicide drug-intoxication death as suicide rather than accident or undetermined, adjusting for region and type of state death investigation system (odds ratio, 1.062; 95% CI,1.016–1.110). Region, too, was a significant predictor. Relative to the South, a 10% increase in drug citation was associated with 43% (95% CI,11%-83%), 41% (95% CI,7%-85%), and 33% (95% CI,1%-76%) higher odds of a suicide classification in the West, Midwest, and Northeast, respectively.

**Conclusion:**

Large interstate variation in the relative magnitude of nonhomicide drug-intoxication deaths classified as suicide by medical examiners and coroners in the US appears partially an artifact of geographic region and degree of toxicological assessment in the case ascertainment process. Etiologic understanding and prevention of drug-induced suicides and other drug-intoxication deaths first require rigorous standardization involving accurate concepts, definitions, and case ascertainment.

## Introduction

Official vital statistics indicate that suicide surpassed motor vehicle traffic crashes as the leading cause of injury mortality in the United States (US) in 2009 [1]. However, this shift may actually have occurred several years earlier, even while it remained undetected. The rate of pharmaceutical and other drug-intoxication deaths rose by 125% between 2000 and 2013 [2], with most being classified as accident (unintentional injury) or undetermined intent (hereafter “undetermined”). Many of these deaths were likely misclassified suicides [3–5]. Suicide is plausibly the most underestimated manner of death in both clinical medicine and public health, since it likely is often obfuscated by death investigations that are inadequate for validly differentiating manner [6,7]. The 21^st^-century drug poisoning epidemic [8, 9], linked with the poor or inadequate quality of determinations of manner among fatal prescription or illicit drug poisoning cases [10,11], poses an important public health hazard and challenge. Without valid data on who has died and the circumstances, it is impossible to identify risks and risk-groups accurately, and thus design, target, and evaluate interventions appropriately. Moreover, nonrandomness in ascertainment of drug-intoxication suicides, in particular, predictably stemming from heterogeneity in death investigation procedures and practices, would generate an artifactual element that could distort cause and manner-of-death rate comparisons between geopolitical units, such as states and counties.

Undetermined is regarded internationally as the manner-of-death category most prone to contain misclassified suicides [12–16]. Poisoning appears much more susceptible to suicide undercounting than the two other leading and more overt and active suicide methods, firearm trauma and hanging/asphyxiation [17–20]. A US study suggests that for drug and other poisoning deaths, the accident (unintentional injury) category may falsely include many more suicides annually than undetermined, given its much larger absolute numbers [4], as well as guidance for professional organizations recommending that utilization of the undetermined category be reserved for those rare cases where available evidence could support more than one manner-of-death determination [21]. Based on an intensive psychiatric evaluation of coroner court records, a postmortem study from England found increased suicide undercounting due to misclassification of pharmaceutical drug-intoxication suicides as accidents [5]. Results from other international studies reveal that the presence of substance abuse in a personal history reduces rather than increases the likelihood that a death will be accurately determined by a medical examiner or a coroner as suicide versus a so-called “accident” [22–24]. They invoke consideration of repeated drug use (as exemplified by needle marks, and evidence of doctor and/or pharmacy shopping from prescription drug monitoring programs) and tolerance (exemplified by multiple refills over a long duration) [21]. These results are buttressed by indirect evidence from one Australian [25] and two US multivariable, multiple-cause-of-death studies [26,27].

Germane to undercounting, standards tend to be very stringent for supporting a determination of suicide in the US and other democratic, higher-income countries [28–30]. A suicide manner-of-death determination, in principle, requires that a medical examiner or coroner both affirm that the mechanism of death was self-inflicted and the decedent intended to die. These conjoined criteria are highly conducive to conservative interpretation. Factors depressing the sensitivity or true-positivity of suicide certification include social stigma, punitive life insurance policy provisions, lack of psychiatric and psychological input into manner-of-death determinations, lack of reliable witness testimony, low autopsy rate, and training deficits among death investigators [3,31,32]. In short, a suicide determination is not a default option for medical examiners and coroners.

A recent descriptive study, covering the period 2008–2010, revealed large interstate differences in the distribution of fatal drug intoxications across the homicide, suicide, accident, and undetermined manner-of-death categories, and in the citation or documentation of one or more specific drugs on the death certificate [33]. The homicide component was negligible, comprising less than 1% of drug-intoxication deaths. Study investigators viewed variation in the relative magnitude of the undetermined category as indicating differential susceptibility of states to undercount drug-intoxication accident deaths in addition to suicides. Undetermineds ranged from 1% of all drug-intoxication deaths in Wyoming to 85% in Maryland, and were less than 5% in 11 states and 15% or higher in 8. Citation of one or more specific drugs on the death certificate in drug-intoxication deaths ranged from 34.8% in Louisiana to 99.4% in West Virginia.

The large variation across US states in drug citation, and the distribution of drug-intoxication deaths classified as suicide, accident, or undetermined, motivated us to evaluate associations between the scope and quality of toxicological testing and type of medicolegal death investigation system, respectively, and the odds that a state would classify a nonhomicide drug-intoxication death as suicide versus accident or undetermined. Representing an empirical backdrop for our analyses, we first reported interstate nonhomicide drug-intoxication death rates by manner of death, as well as state and regional comparisons on the outcome variable, the relative magnitude of drug-intoxication suicides. Our general research question may be restated as follows: Is interstate variation in the relative magnitude of nonhomicide drug-intoxication deaths classified as suicide by medical examiners and coroners a partial artifact of heterogeneity in toxicology and death investigation systems?

In this national, state-based, ecological study, we tested the hypothesis that the odds a state would classify a nonhomicide drug-intoxication death as suicide, versus accident or undetermined, vary positively with citation of one or more specific drugs on the death certificate. We then assessed whether type of state death investigation system was associated with the outcome variable. Embedded in this research question was our second and final hypothesis: a decentralized county coroner state has lower odds than a centralized medical examiner state of classifying a nonhomicide drug-intoxication death as suicide, versus accident or undetermined. We posit that decentralized county coroner systems tend to have the least forensic expertise and resources, the least uniform death investigation protocols, the least standardized measurement, the least quality control [34], and are the most sociopolitically vulnerable or inhibited in determining suicide, as a highly stigmatized phenomenon [35,36].

Although the rare coroner system functions as a medical examiner system, these two systems generally exhibit disparate philosophical underpinnings and forensic approaches in their death investigations [7,37]. Governed by variable standards, such as “more likely than not” or “preponderance of evidence,” medical examiners and coroners respectively diagnose or rule a given death as a suicide. Medical examiners are appointed, and are usually physicians with training in pathology and forensic pathology, and board certification in both disciplines. They may serve more than one county and often an entire state. By contrast, coroners are lay county-level officials, who are generally elected and have limited if any medical background. Whereas some coroners may employ certified forensic pathologists in their offices, this is normally not a requirement. Coroners typically have less experience with toxicological terminology than do medical examiners, and most often, less contact with medical and forensic toxicologists [33]. Furthermore, they likely feel greater budgetary constraints when considering whether to obtain toxicological tests, and in conducting forensic death investigations as a whole. Reflecting this likelihood, only 62% of decentralized county coroner states specified at least one drug in certifying their drug-intoxication deaths during the period, 2008–2010, versus 92% of centralized medical examiner system states, 73% of states with a mix of decentralized coroner and medical examiner systems, and 71% of decentralized medical examiner system states.

Selected *a priori*, we factored into our study five state-level sociodemographic covariates, whose absence might confound assessment of the hypothesized relationships concerning toxicological scope and quality and the relative forensic sophistication of state death investigation systems. They were age distribution, gender ratio, urbanization rate, poverty rate, and region. Age and gender show an association with potential suicide misclassification by medical examiners and coroners at the individual level [19]. Urbanization and poverty have implications for whether there is a critical mass of expertise and resources to support high-quality medicolegal death investigations, independent of death investigation system type. Our fifth sociodemographic covariate, region, was an important consideration because of striking historical differences in suicide rates between the West and South relative to the Northeast [38], and potential regional variation in suicide misclassification [39].

## Methods

The principal data source was the Multiple Cause-of-Death public use files, for years 2008–2010, produced by the National Center for Health Statistics (NCHS) (accessible at http://webappa.cdc.gov/sasweb/ncipc/mortrate10_us.html). Causes of death were coded under the *International Statistical Classification of Diseases and Related Health Problems*, Tenth Revision (ICD-10^th^) [40]. In this national ecological mortality study, we included all 111,583 fatalities having an underlying cause of death of drug intoxication, with manner of death coded as suicide (X60-64), accident (X40-44), or undetermined (Y10-14). Underlying cause in this context represents a death determined by a medical examiner or coroner to be caused by drug intoxication, and is first coded by intent, that is, accident, suicide, or undetermined, and then by cause-specific drug groups corresponding to our selected ICD-10^th^ manner-of-death codes: nonopioid analgesics (X40, X60, Y10), sedative hypnotics (X41, X61, Y11), narcotics (X42, X62, Y12), other autonomic nervous system drugs (X43, X63, Y13), and other and unspecified drugs (X44, X64, Y14). Homicides were excluded from the analyses, since they comprised less than 1% of drug-intoxication deaths. Our unit of analysis was the state, and we incorporated all 50 states into the study. We annualized the three-year rates to stabilize the mortality data, based on official total counts, but also assessed remaining sampling variability.

We adopted our observation period and the two analytic variables, citation of one or more specific drugs on the death certificate and type of state death investigation system, from the previously documented interstate drug-intoxication mortality study [33]. Drug citation indexed the scope and quality of the toxicological evaluation. Type of death investigation system comprised three categories representing a putative hierarchy of forensic sophistication across states [7]. At the top were centralized medical examiner system states, followed by a combined category of decentralized medical examiner system states and states comprising a hybrid or mix of decentralized county coroner and medical examiner systems. Decentralized county coroner system states constituted the base. The three categories represented 16, 23, and 11 states, respectively. Composition of our intermediate category was justified by similar drug citation prevalence and the small number of decentralized medical examiner states (6) [33]. In a minor departure from source categorization, we reclassified North Dakota from a decentralized county coroner state to a hybrid system state because of the active roles that the State Forensic (Medical) Examiner’s office and the University of North Dakota fulfill in death investigations in parts of that state [According to emails: Randy L. Hanzlick, MD (October 23, 2014), William Massello III, MD (October 24, 2014) and Mary Ann Sens, MD (October 27, 2014)].

We operationalized our sociodemographic covariates as follows: age distribution as an annual average percentage of the civilian, non-institutional population for 2008–2010 for age groups 0–14, 15–64, and 65 years and older; gender ratio as the annual average number of males per 100 females for 2008–2010 (the data for the preceding two variables were accessed at http://webappa.cdc.gov/sasweb/ncipc/dataRestriction_inj.html); urbanization rate as the percentage of state population that resided in urban areas according to the 2010 national Census (accessible at http://www.census.gov/prod/2011pubs/12statab/labor.pdf); poverty rate as the annualized percentage of people in a state living in poverty according to thresholds utilized in the 2008–2010 American Community Surveys (accessible at http://www.census.gov/prod/2010pubs/acsbr09-1.pdf and http://www.census.gov/prod/2012pubs/acsbr11-01.pdf), and region as a four-category Census classification: West/Midwest/Northeast/ South (accessible at http://webappa.cdc.gov/sasweb/ncipc/dataRestriction_inj.html).

The outcome variable was the proportion of suicides among drug-intoxication deaths, defined as $p=\frac{suicide}{suicide+undetermined+accident}$. ArcGIS 10 was used to map these data by state and region (Esri, Redlands, CA). The relative magnitude of suicides among drug-intoxication deaths was assumed to vary inversely with the proneness of states to misclassify these suicides, with the accident and undetermined manner-of-death categories being the major reservoirs for the misclassifications [3]. We used a logit-transformed model, $log\left(\frac{p}{1-p}\right)=\beta_{0}+\beta_{1}log\left(drug\;citation\right)+\beta_{2}system,$ in computing the odds ratio (OR) for a drug-intoxication death being classified as suicide, versus accident or undetermined: OR = *e*.^*β*^ The multivariable analysis followed a backwards elimination linear regression process, with type of state death investigation system locked into the model. Covariate selection emanated from optimization of the Bayes Information Criterion (BIC).

Preceding the regression analyses, we computed a Spearman rank-order correlation coefficient to assess the congruence of interstate drug-intoxication suicide rates with corresponding combined accident and undetermined drug-intoxication death rates. We assumed that a low correlation would indicate variable potential of states to account appropriately for drug-intoxication suicides. The statistical analyses were performed using SAS V9.3 (SAS Institute, Cary, NC).

## Results

**Fig 1:**
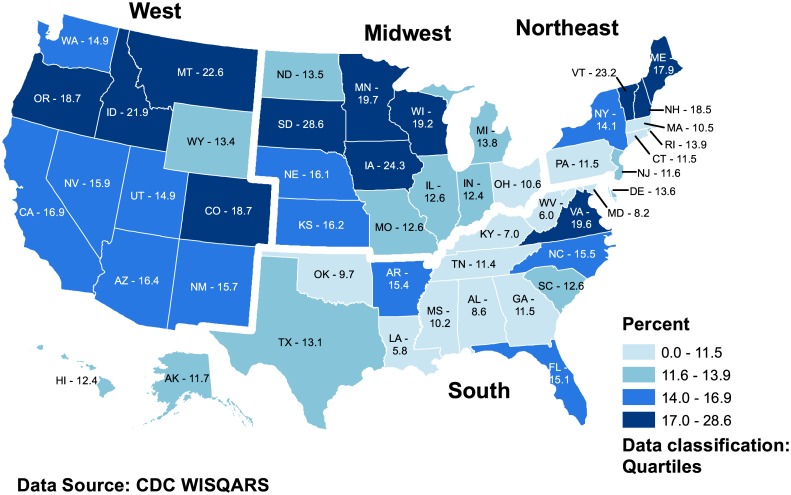
Percentage of nonhomicide drug-intoxication deaths classified as suicides in quartiles by state and region, United States, 2008–2010.

During the observation period, 2008–2010, drug-intoxication death rates ranged from 4.8 per 100,000 population in North Dakota to 23.4 in New Mexico (Table 1). Drug-intoxication suicide rates varied between 0.6 per 100,000 in North Dakota and 3.7 in New Mexico. A rank-order comparison showed a low correlation across states between their drug-intoxication suicide rates and corresponding combined drug-intoxication accident and undetermined rates. The percentage of nonhomicide drug-intoxication deaths classified as suicide, versus accident or undetermined, ranged from 6% in Louisiana to 29% in South Dakota (Fig 1). Percentages peaked in the West and Midwest, and were lowest in Oklahoma, Massachusetts, Connecticut, and a corridor extending from Louisiana, Mississippi, Alabama, and Georgia in the South through to Ohio, Maryland, Pennsylvania, and New Jersey.

**Table 1:** Annualized rates for selected cause-specific manners of death and specific drug citation by state/death investigation system type: United States, 2008–2010.

State/Death Investigation System Type	Nonhomicide Drug-intoxication Death Rates by Manner (per 100,000)^a^				Drug-intoxication Deaths with 1+ Specific Drugs Cited on the Death Certificate (%)^b^
	Suicide	Undetermined	Accident	Total	
**All States**	**1.7**	**1.0**	**9.5**	**12.2**	**75.1%**
**Centralized medical examiner**					
Alaska	1.9	1.7	12.7	16.3	95.5
Connecticut	1.2	0.5	8.9	10.7	76.8
Delaware	2.1	1.3	1.8	15.1	79.3
Maine	2.1	0.3	9.3	11.8	89.7
Maryland	1.0	9.9	1.2	12.1	98.6
Massachusetts	1.3	0.6	10.1	12.0	97.0
New Hampshire	2.1	0.9	8.5	11.5	99.1
New Mexico	3.7	1.2	18.5	23.4	68.7
North Carolina	1.9	0.5	9.8	12.3	92.9
Oklahoma	1.8	1.1	15.3	18.3	97.2
Oregon	2.4	1.4	9.0	12.7	91.2
Rhode Island	2.3	0.3	13.6	16.2	97.3
Utah	2.5	6.6	7.8	16.9	94.2
Vermont	2.3	0.9	6.9	10.1	98.9
Virginia	1.6	0.3	6.3	8.2	92.7
West Virginia	1.3	1.9	18.4	21.5	99.4
**Decentralized county coroner**					
Arkansas	1.9	2.9	7.4	12.2	76.7
Colorado	2.7	1.3	10.5	14.6	70.4
Idaho	2.3	1.7	6.6	10.6	59.6
Indiana	1.7	2.2	9.7	13.7	45.8
Kansas	1.5	0.9	6.9	9.3	58.8
Louisiana	0.8	1.6	11.1	13.5	34.8
Nebraska	0.9	0.6	4.3	5.8	69.4
Nevada	3.3	0.6	16.6	20.4	97.7
South Carolina	1.7	0.2	11.6	13.5	59.8
South Dakota	1.7	1.2	3.1	6.1	88.8
Wyoming	1.8	0.8	10.8	13.4	64.8
**Decentralized medical examiner (DME) or combined county coroner/medical examiner**					
Alabama	1.1	1.0	10.4	12.5	45.0
Arizona (DME)	2.5	1.2	11.7	15.4	80.2
California	1.8	0.4	8.5	10.8	73.1
Florida (DME)	2.5	0.5	13.3	16.3	68.8
Georgia	1.2	0.4	8.7	10.4	71.4
Hawaii	1.3	2.0	6.9	10.1	83.1
Illinois	1.3	0.4	8.7	10.4	86.5
Iowa (DME)	1.8	0.7	4.8	7.4	96.1
Kentucky	1.4	1.7	16.6	19.6	64.8
Michigan (DME)	1.9	2.2	9.5	13.6	65.8
Minnesota	1.5	0.9	5.1	7.6	82.4
Mississippi	1.1	1.1	8.6	10.8	43.4
Missouri	1.8	0.8	12.0	14.6	79.3
Montana	3.0	1.8	8.5	13.4	69.9
New Jersey (DME)	0.8	0.2	5.9	7.0	59.3
New York	1.2	0.7	6.5	8.4	94.0
North Dakota	0.6	0.2	4.0	4.8	87.9
Ohio	1.5	0.6	11.8	13.9	71.4
Pennsylvania	1.7	0.7	12.4	14.9	45.0
Tennessee (DME)	1.8	1.2	12.8	15.7	77.8
Texas	1.2	0.3	7.7	9.2	74.7
Washington	2.2	0.9	11.5	14.6	92.6
Wisconsin	2.1	0.8	8.1	11.0	85.9

^a^ Spearman’s rank-order correlation coefficient (Rho) for drug-intoxication suicide rates and combined accident and undetermined intent drug-intoxication death rates = 0.38 (p<0.01).

^b^ Includes drug-intoxication homicides. Source: Warner M, Paulozzi LJ, Nolte KB, Davis GG, Nelson LS. State variation in certifying manner of death and drugs involved in drug-intoxication deaths. *Acad Forensic Pathol*. 2013; 3(2): 231–237.

The multivariable analysis showed that citation of one or more specific drugs on the death certificate was positively associated with the odds that a state would classify a nonhomicide drug-intoxication death as a suicide, versus accident or undetermined, adjusting for type of state death investigation system and region (p = 0.024) (Table 2). We projected that a relative 10% increase in drug citation (such as from 50% to 55%), for example, would increase the odds of the death being classified as suicide by 6.2%: (1+10%)^*β*^ − 1 = 1.1^0.630^ − 1 = 0.062. Of note, our death investigation system hypothesis was not empirically supported (p = 0.210).

**Table 2:** Predicting the odds of a nonhomicide drug-intoxication death being classified as suicide versus accident or undetermined intent.

**Single Risk Factor Model**			**95% CI**		
	**Beta**	**Odds Ratio**	**Lower**	**Upper**	**Prob**
**Drug citation** ^**a**^	0.526	1.051	1.011	1.093	0.015
**Region** ^**b**^					
West	0.460	1.584	1.234	2.035	0.001
Midwest	0.448	1.565	1.212	2.021	0.001
Northeast	0.313	1.368	1.035	1.809	0.033
South (referent)		1.000			
**Death investigation system type**					
Decentralized county coroner	0.128	1.137	0.841	1.537	0.409
Combined^**c**^	-0.007	0.993	0.773	1.275	0.954
Centralized medical examiner (referent)		1.000			
**Multivariable Model**			**95% CI**		
	**Beta**	**Odds Ratio**	**Lower**	**Upper**	**Prob**
**Drug citation** ^**a**^	0.630	1.062	1.016	1.110	0.011
**Region**					
West	0.358	1.430	1.115	1.836	0.007
Midwest	0.341	1.407	1.072	1.846	0.018
Northeast	0.289	1.335	1.014	1.756	0.045
South (referent)		1.000			
**Death investigation system type**					
Decentralized county coroner	0.298	1.347	0.956	1.899	0.096
Combined^**c**^	0.104	1.110	0.852	1.446	0.444
Centralized medical examiner (referent)		1.000			

^a^ Assuming a 10% increase in citation of 1 or more specific drugs on the death certificate.

^b^ Age, gender, urbanization, and poverty, the 4 other pre-selected sociodemographic covariates were not significant predictors of suicide classification.

^c^ Comprises decentralized medical examiner states and states with hybrid county coroner/medical examiner systems.

Assuming a 10% increase in citation of one or more specific drugs on the death certificate, geographic region emerged as the only one of our five pre-selected sociodemographic covariates to predict the relative odds of a suicide classification, with adjustment for drug citation and type of death investigation system (p = 0.024). Collectively by region, western, midwestern, and northeastern states respectively showed 43%, 41%, and 33% higher odds than southern states of classifying a nonhomicide drug-intoxication death as suicide. There was no interaction between region and type of state death investigation system (p = 0.965).

## Discussion

One of our two research hypotheses was affirmed; namely, that there was a positive association between citation of one or more specific drugs on the death certificate in states and classification by a state of a nonhomicide drug-intoxication death as suicide—not a default option—versus accident or undetermined. However, centralized medical examiner states showed no statistically significant difference from decentralized county coroner system states in their odds of suicide classification. Whereas centralized medical examiner states manifested a much higher mean citation of one or more specific drugs on the death certificate (92% versus 62%) than did decentralized county coroner states [33], the values for the latter were more widely dispersed across the citation continuum (Table 1).

Region, a sociodemographic covariate, was the predominant predictor of a suicide classification among nonhomicide drug-intoxication deaths. Mixed-methods research will be necessary to illuminate the gulf between the South and the other three major geographic regions. Explanations will likely be sociocultural, political, and economic, as well as forensic. Additional research questions emanate from the relatively low proportion of suicides among drug-intoxication deaths in some centralized medical examiner states, such as West Virginia, Maryland, Oklahoma, Massachusetts, and Connecticut, the relatively high proportion in the decentralized county coroner states of South Dakota, Idaho, Colorado, Kansas, and Nevada, and the sharp divide in the magnitude of the relative proportions that separate northern and southern New England. Our results reinforce national concerns about the resourcing and quality of forensic death investigations in much of the country, irrespective of system type [7]. These concerns are informed by a perspective that focuses on public health problems [41], such as suicide, rather than one which emphasizes homicide and associated criminal justice obligations [7].

Compared to medical examiner systems, county coroner systems are disproportionately located in more rural and less affluent areas [37]. Coroner offices comprise approximately 68% of the medicolegal death investigation offices nationwide, but a majority serve populations of less than 25,000 [7]. A recent document, prepared for the National Commission on Forensic Science, reported that fewer than 100 of the estimated 2,479 death investigation offices are accredited by either the National Association of Medical Examiners (NAME), approximately 70, or the International Association of Coroners and Medical Examiners (IAC & ME), approximately 25 [42]. Both organizations have instituted rigorous evaluative processes, and salient professional organizations accept the respective accreditations as conformance to national standards. However, scarce resources appear to be impeding the external recognition of these processes that would signal compliance with established international standards. Moreover, accredited offices fall well short of covering the preponderance of the national population.

Our ecological study has several limitations. Suicides are local phenomena, even as responsibility for coding and mortality record-keeping extends to the state, with states then sharing their accumulated data with NCHS. Since the state was the analytic unit, our sample size was small. This choice precluded us from examining the impact of the accreditation process and district and county-level heterogeneity in investigative procedures and practices on the relative magnitude of suicides among drug-intoxication deaths, including whether elected and appointed lead officials differ in their approaches to suicide determination. Reference to “abuse,” “addiction,” or “misuse” on the death certificate may induce nonrandom categorization of drug-intoxication fatalities, including possible suicides, as mental disorders under ICD across sociodemographic groups and types of state death investigation systems [21]. Thus, also a potential impediment in addressing our research questions, the missed suicides would be differentially susceptible to misclassification under natural causes, the sole disease manner-of-death category.

In addition, the study lacked comparative background data on medical examiners and coroners, their resources, and competing demands. Another research limitation is that citation of one or more specific drugs on the death certificate was a proxy for the scope and quality of a toxicological evaluation. Moreover, our approach was necessarily indirect, given the logistical and economic infeasibility of comprehensively reviewing thousands of death records nationwide to determine the extent and variation in undercounting drug-intoxication suicides across all states. Nevertheless, the research limitations do not mitigate the implications of our findings that susceptibility of states to undercount drug-intoxication suicides varies inversely with the scope and quality of toxicological evaluations, and that such undercounting is most prevalent in the South. The creation of a national surveillance model for drug-poisoning deaths, which includes Federal support for uniform toxicological testing in these cases, would likely yield a more accurate assessment of suicides across the states. Such a model could be used to guide public health interventions.

US mortality rates for both total suicide and lethal drug intoxication, in the combined suicide, undetermined, and accident manners-of-death component, have burgeoned during the opening years of the 21st century [1,4,8]. Most of the drug-intoxication deaths apparently reflect deliberate use by the decedents of prescribed compounds, street-diverted opiate medications, and illicit compounds (e.g., heroin) [43], often in combination with alcohol [3]. But a major unknown is how many were intended to be lethal, how many involved deliberate ingestion for the purposes of intoxication but not suicide, and how many were truly “accidental” or unintentional. Once suicide is excluded as a manner of death, due to an apparent lack of intent, most drug-intoxication deaths are predominantly classified as an “accident” or “unintentional.” Typically they are not true accidents, however, since they arise from deliberate, motivated behaviors that predictably increase the probability of death. Hence the accident category includes both misclassified suicides and many deaths where deliberate behaviors were the cause, even if there were no intention to die on the day of death. This misclassification and misconceptualization of drug-intoxication deaths, which belong on the upper end of a self-harm continuum [44], provided us with the impetus to propose a more appropriate grouping of drug deaths to enable researchers, prevention scientists, and public health practitioners to circumvent their dependence upon extant manner-of-death classification.

In collaboration with other members of a multidisciplinary team, we recently proposed that most nonhomicide drug-intoxication deaths should first be codified under a new rubric labeled “death from drug self-intoxication” (DDSI) [3]. Employing an etiologic, surveillance, and prevention perspective, we conceived DDSI as an objective characterization of premorbid behavior that would transcend epidemiologic coding restrictions inherent both in the current medicolegal paradigm and the problematic ascertainment of drug-intoxication suicides by medical examiners and coroners. To facilitate national, state, and county accounting and prevention of DDSIs, a related and critical intervention would be a standardized provision on all death certificates for evidence-based documentation of premorbid drug misuse and abuse. More generally, integrating the accounting of all-method suicides with nonsuicide drug-intoxication deaths, which were unintended outcomes of hazardous, self-injurious behaviors, into an overarching category of self-injury mortality is essential for effective clinical and public health policy and practice [3,44,45].

## Conclusion

Large interstate variation in the relative magnitude of nonhomicide drug-intoxication deaths classified by medical examiners and coroners as suicide in the US appears partially an artifact of geographic region and degree of toxicological assessment in the case ascertainment process. Etiologic understanding and prevention of drug-induced suicides and other drug-intoxication deaths first require rigorous standardization involving accurate concepts, definitions, and case ascertainment.
