# Correction: Variable Classification of Drug-Intoxication Suicides across US States: A Partial Artifact of Forensics?

**DOI:** 10.1371/journal.pone.0137933

**Published:** 2015-09-03

**Authors:** Ian R. H. Rockett, Gerald R. Hobbs, Dan Wu, Haomiao Jia, Kurt B. Nolte, Gordon S. Smith, Sandra L. Putnam, Eric D. Caine

Table 1 appears incorrectly in the published article. Please view the correct Table 1 here.

**Table 1:** Annualized rates for selected cause-specific manners of death and specific drug citation by state/death investigation system type: United States, 2008–2010

State/Death Investigation System Type	Nonhomicide Drug-intoxication Death Rates by Manner (per 100,000)^a^				Drug-intoxication Deaths with 1+ Specific Drugs Cited on the Death Certificate (%)^b^
	Suicide	Undetermined	Accident	Total	
All States	1.7	1.0	9.5	12.2	75.1%
**Centralized medical examiner**					
**Alaska**	**1.9**	**1.7**	**12.7**	**16.3**	**95.5**
**Connecticut**	**1.2**	**0.5**	**8.9**	**10.7**	**76.8**
**Delaware**	**2.1**	**1.3**	**1.8**	**15.1**	**79.3**
**Maine**	**2.1**	**0.3**	**9.3**	**11.8**	**89.7**
**Maryland**	**1.0**	**9.9**	**1.2**	**12.1**	**98.6**
**Massachusetts**	**1.3**	**0.6**	**10.1**	**12.0**	**97.0**
**New Hampshire**	**2.1**	**0.9**	**8.5**	**11.5**	**99.1**
**New Mexico**	**3.7**	**1.2**	**18.5**	**23.4**	**68.7**
**North Carolina**	**1.9**	**0.5**	**9.8**	**12.3**	**92.9**
**Oklahoma**	**1.8**	**1.1**	**15.3**	**18.3**	**97.2**
**Oregon**	**2.4**	**1.4**	**9.0**	**12.7**	**91.2**
**Rhode Island**	**2.3**	**0.3**	**13.6**	**16.2**	**97.3**
**Utah**	**2.5**	**6.6**	**7.8**	**16.9**	**94.2**
**Vermont**	**2.3**	**0.9**	**6.9**	**10.1**	**98.9**
**Virginia**	**1.6**	**0.3**	**6.3**	**8.2**	**92.7**
**West Virginia**	**1.3**	**1.9**	**18.4**	**21.5**	**99.4**
**Decentralized county coroner**					
**Arkansas**	**1.9**	**2.9**	**7.4**	**12.2**	**76.7**
**Colorado**	**2.7**	**1.3**	**10.5**	**14.6**	**70.4**
**Idaho**	**2.3**	**1.7**	**6.6**	**10.6**	**59.6**
**Indiana**	**1.7**	**2.2**	**9.7**	**13.7**	**45.8**
**Kansas**	**1.5**	**0.9**	**6.9**	**9.3**	**58.8**
**Louisiana**	**0.8**	**1.6**	**11.1**	**13.5**	**34.8**
**Nebraska**	**0.9**	**0.6**	**4.3**	**5.8**	**69.4**
**Nevada**	**3.3**	**0.6**	**16.6**	**20.4**	**97.7**
**South Carolina**	**1.7**	**0.2**	**11.6**	**13.5**	**59.8**
**South Dakota**	**1.7**	**1.2**	**3.1**	**6.1**	**88.8**
**Wyoming**	**1.8**	**0.8**	**10.8**	**13.4**	**64.8**
**Decentralized medical examiner (DME) or combined county coroner/medical examiner**					
**Alabama**	**1.1**	**1.0**	**10.4**	**12.5**	**45.0**
**Arizona (DME)**	**2.5**	**1.2**	**11.7**	**15.4**	**80.2**
**California**	**1.8**	**0.4**	**8.5**	**10.8**	**73.1**
**Florida (DME)**	**2.5**	**0.5**	**13.3**	**16.3**	**68.8**
**Georgia**	**1.2**	**0.4**	**8.7**	**10.4**	**71.4**
**Hawaii**	**1.3**	**2.0**	**6.9**	**10.1**	**83.1**
**Illinois**	**1.3**	**0.4**	**8.7**	**10.4**	**86.5**
**Iowa (DME)**	**1.8**	**0.7**	**4.8**	**7.4**	**96.1**
**Kentucky**	**1.4**	**1.7**	**16.6**	**19.6**	**64.8**
**Michigan (DME)**	**1.9**	**2.2**	**9.5**	**13.6**	**65.8**
**Minnesota**	**1.5**	**0.9**	**5.1**	**7.6**	**82.4**
**Mississippi**	**1.1**	**1.1**	**8.6**	**10.8**	**43.4**
**Missouri**	**1.8**	**0.8**	**12.0**	**14.6**	**79.3**
**Montana**	**3.0**	**1.8**	**8.5**	**13.4**	**69.9**
**New Jersey (DME)**	**0.8**	**0.2**	**5.9**	**7.0**	**59.3**
**New York**	**1.2**	**0.7**	**6.5**	**8.4**	**94.0**
**North Dakota**	**0.6**	**0.2**	**4.0**	**4.8**	**87.9**
**Ohio**	**1.5**	**0.6**	**11.8**	**13.9**	**71.4**
**Pennsylvania**	**1.7**	**0.7**	**12.4**	**14.9**	**45.0**
**Tennessee (DME)**	**1.8**	**1.2**	**12.8**	**15.7**	**77.8**
**Texas**	**1.2**	**0.3**	**7.7**	**9.2**	**74.7**
**Washington**	**2.2**	**0.9**	**11.5**	**14.6**	**92.6**
**Wisconsin**	**2.1**	**0.8**	**8.1**	**11.0**	**85.9**

^a^ Spearman’s rank-order correlation coefficient (Rho) for drug-intoxication suicide rates and combined accident and undetermined intent drug-intoxication death rates = 0.38 (p<0.01).

^b^ Includes drug-intoxication homicides. Source: Warner M, Paulozzi LJ, Nolte KB, Davis GG, Nelson LS. State variation in certifying manner of death and drugs involved in drug-intoxication deaths. *Acad Forensic Pathol*. 2013; 3(2): 231–237.

## References

[1] Rockett IRH, Hobbs GR, Wu D, Jia H, Nolte KB, Smith GS, et al (2015) Variable Classification of Drug-Intoxication Suicides across US States: A Partial Artifact of Forensics? PLoS ONE 10(8): e0135296 doi:10.1371/journal.pone.0135296 26295155 10.1371/journal.pone.0135296 PMC4546666

